# Chitosan-Based Films Containing Rutin for Potential Cosmetic Applications

**DOI:** 10.3390/polym15153224

**Published:** 2023-07-28

**Authors:** Alina Sionkowska, Katarzyna Lewandowska, Marzanna Kurzawa

**Affiliations:** 1 Department of Biomaterials and Cosmetic Chemistry, Faculty of Chemistry, Nicolaus Copernicus University in Torun, Gagarin 7 Street, 87100 Torun, Poland; reol@umk.pl; 2 Department of Analytical Chemistry and Applied Spectroscopy, Faculty of Chemistry, Nicolaus Copernicus University in Torun, Gagarin 7 Street, 87100 Torun, Poland; jmk@umk.pl

**Keywords:** chitosan, rutin, cosmetics, skin

## Abstract

Chitosan is a polysaccharide with film-forming properties. Such properties are widely used for the preparation of beauty masks and wound-healing materials. In this work, chitosan-based films containing hyaluronic acid and rutin have been researched for potential cosmetic applications. Rutin was added to a chitosan solution in lactic acid, and then thin films were fabricated. The structure of the films was studied using FTIR spectroscopy. Surface properties were studied using an AFM microscope. The release of rutin from chitosan-based film was researched by the HPLC method. The properties of the skin, such as elasticity and moisturization, were studied using the Aramo TS 2 apparatus. It was found that the addition of rutin did not have an influence on the chitosan structure but affected its thermal stability. The roughness of the films was bigger after the addition of rutin to chitosan-based films. Skin elasticity and skin moisturization were somewhat improved after the topical application of the proposed chitosan–rutin mask. The maximum release of rutin was found after 20 min at pH 5.5, related to the pH of normal human skin. The average percentage of release from chitosan-based film containing hyaluronic acid was smaller than from chitosan-based films.

## 1. Introduction

Chitosan is a biopolymer obtained from chitin in the deacetylation process. Chitin is structurally related to the most widespread natural polysaccharide—cellulose, but the difference is in the C2 position due to the presence of the acetamide group. Chitosan is a copolymer made of two structural units: N-acetyl-D-glucosamine and D-glucosamine [1,2]. Chitosan is recognized as a sustainable polymer characterized by the degree of deacetylation (DDA) and high molecular weight [2,3]. The commercially available chitosan is usually of marine origin and is obtained by a chemical process. However, scientists are working on alternative biotechnological processes of production, such as enzymatic methods, including fermentation. Chitosan can also be obtained from alternative sources, including insects and fungi. 

Chitosan is widely used in several fields [4,5,6,7,8,9,10,11,12]. It can be used in biomedical fields [4,5,6,7,8] and cosmetic formulations [8,9,10,11,12,13,14]. The reason for wide chitosan applications is its anti-microbial properties [15]. The above-mentioned properties can be very useful in food packaging [16,17,18,19,20]. The review of cosmetic applications of chitosan has been done by us recently [8]. Chitosan is mainly used in cosmetic formulation due to its film-forming and hair-fixing properties. Film-forming properties are often used for the preparation of cosmetic masks and wound dressings [21,22,23,24,25,26,27,28,29]. Chitosan-based materials are also widely used in the delivery of drugs and active compounds [30,31,32,33,34]. The properties of chitosan-based materials depend on the molecular weight of the polymer and the degree of deacetylation. For chitosan with a high degree of deacetylation and a high number of unsubstituted amino groups, an increase in antioxidant activity was observed, whereas high average molecular weight reduces this activity [8]. In the cosmetics industry, there is a constant need for new derivatives of chitosan because chitosan itself is not soluble in water. The most significant chitosan derivatives used in cosmetics are carboxymethyl chitosan (CMCh), N-succinoyl chitosan (NSCS), partially myristoylated carboxymethyl chitosan (PMCC), chitosan lactate (CL), and chitosan acetate (CAc). Moreover, chitosan and its derivatives can be cross-linked to obtain a three-dimensional polymer network. The cross-linking effects can be achieved by the use of chemical or physical agents [8]. The physical methods of chitosan modification include cross-linking using gamma rays or photo cross-linking with UV radiation. Chemical cross-linking of chitosan usually uses glutaraldehyde (GA), genipin, and polyethylene glycol. 

From a cosmetic point of view, it is crucial to incorporate active substances into chitosan matrices. There are several examples in the literature about such preparation; for example, chitosan–caffeic acid conjugates have been proposed as material against antibiotic-resistant acne-related bacteria [35], and chitosan has been proposed for the controlled release of vitamin C [36]. Recent developments in chitosan encapsulation of various active ingredients for multifunctional applications have been summarized by Raza et al. [37]. 

In this work, we prepared chitosan-based films containing rutin. Rutin is a polyphenolic flavonoid with anti-inflammatory and antioxidant properties [38].

It is abundantly found in garlic and other food sources [39]. In recent years, a number of studies investigated the diverse biological effects of rutin, such as anti-microbial, anti-carcinogenic, anti-thrombotic, cardioprotective, and neuroprotective activities along with improving the liver’s health, function, and integrity [39,40,41].

Rutin is used in cosmetic dermal products such as anti-aging products and cosmetic preparations for atopic and allergic skin [23,42,43], mainly due to its antioxidative properties [44,45]. However, there is some limitation in its application due to the poor water solubility of rutin. It cannot be used efficiently in dermal products because of its insufficient penetration rate into the skin. The poor penetration of rutin into the skin is a result of its low solubility in both aqueous and organic solvents. For this reason, new ways to increase the penetration of the skin and thus increase the beneficial effect for skin using rutin have been considered. For example, the rutin nano-suspension and nanocrystals have been researched to enhance its penetration [46].

The aim of this research was the preparation of a chitosan-based film containing rutin and a chitosan–hyaluronic acid film containing rutin for potential cosmetic and biomedical applications. Chitosan/HA/rutin films have been tested for their physicochemical properties and skin treatment. Moreover, the release of rutin from biopolymer films was studied by the HPLC method. 

## 2. Materials and Methods

### 2.1. Materials

**Figure 1:**
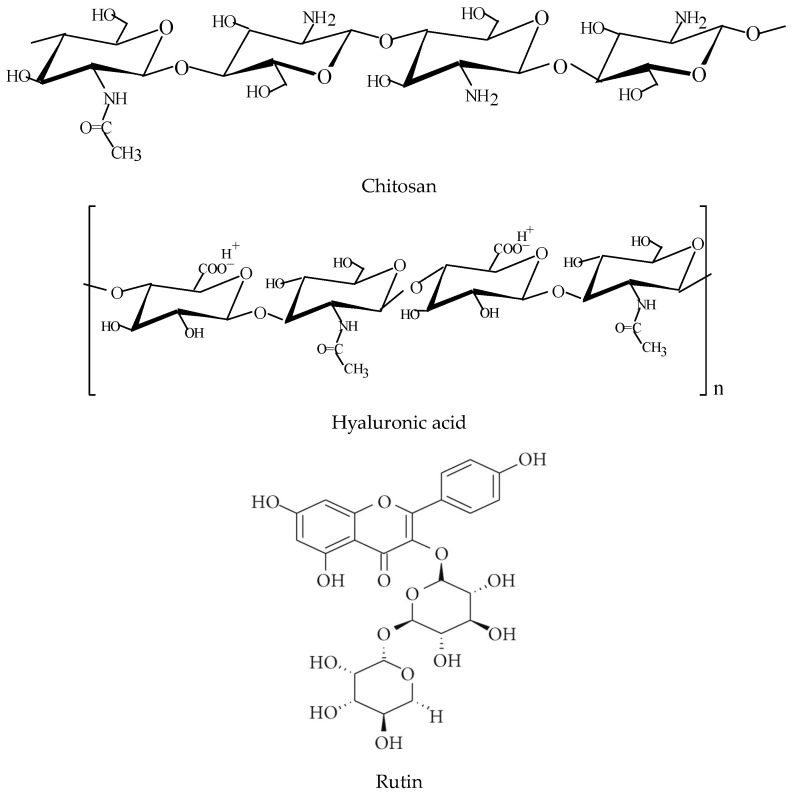
Chemical structures of biopolymers and active substances used in the research.

Chitosan (CS, medium molecular weight 1,113,000 g mol^−1^ and degree of deacetylation 75%), lactic acid (C_3_H_6_O_3_), and rutin hydrate (RUT, C_27_H_30_O_16_xH_2_O) ≥ 95% (HPLC) powder were purchased from Sigma-Aldrich (Poznań, Poland). Glycerin anhydrous pure (GLY, C_3_H_8_O_3_) was purchased from Chempur (Piekary Śląskie, Poland). Hyaluronic acid (HA) was a cosmetic grade, and its structure was confirmed using IR spectra. All reagents were used without further purification. The structures of chemical compounds used in this research are shown in Figure 1. 

### 2.2. Preparation of Polymer Films Modified by Rutin

Chitosan (2% *w*/*v*) was completely dissolved in lactic acid (0.3M) at room temperature for 72 h. Lactic acid (LA) was used to dissolve chitosan. LA is widely used in cosmetic formulation for pH modification. Chitosan itself is not soluble in water, so acidic media was needed to obtain a soluble form of chitosan. The prepared chitosan solution was blended separately with other components, including glycerin, rutin, and hyaluronic acid, forming three types of films CS/GLY, CS/GLY/RUT, and CS/GLY/RUT/HA. The composition of the formulation is shown in Table 1. Glycerin liquid was blended with chitosan solution in a 0.5:1 ratio to that of chitosan powder. Rutin (RUT) powder and hyaluronic acid solution (1%) were added with the ratio of 1% w/w (relative to chitosan) and stirred at 100 rpm for 24 h. To achieve the composite films, a 32 mL polymer solution was poured into the squared Petri dishes (100 × 15 mm) and then allowed to air dry (three days) and subsequently used for further analysis. Film thickness was carried out using a handheld micrometer to the nearest 0.001 mm. Measurements were taken in at least seven random locations of each film, and values were reported as mean ± standard deviation (SD).

### 2.3. Fourier-Transformed Infrared Spectroscopy (FTIR)

FTIR analysis of the modified chitosan films (CS/HA/RUT) has been done using a Nicolet iS10 spectrometer (Thermo Fisher Scientific, Waltham, MA, USA) attached with an attenuated total reflectance mode (iD5-Ge-ATR) assembly. The samples were scanned at a 2.0 cm^−1^ resolution using 100 scans in the wavenumber range of 400–4000 cm^−1^.

### 2.4. Thermal Stability-TA

Thermogravimetric analysis was performed at a heating rate of 20 °C/min (20–600 °C) in the nitrogen atmosphere by using TA Instruments SDT 2960 Simultaneous TGA-DTA (TA Instruments Manufacturer, Eschborn, Germany). From thermogravimetric curves, the characteristic temperature at a maximum decomposition rate of the investigated composites was determined.

### 2.5. AFM and SEM Investigations

The morphology of the prepared films was characterized by an AFM microscope The pictures were obtained by MultiMode Scanning Probe Microscope Nanoscope IIIa (Digital Instruments Veeco Metrology Group, Santa Barbara, CA, USA) operating in the tapping mode, in air, in room temperature.. The roughness of the films was calculated from atomic force microscopy images obtained using the Veeco SPM (digital instrument) microscope and calculated for the scanned area (5 µm × 5 µm) using Nanoscope software. The AFM calculations for biopolymer films were obtained for different places in the film, and the most typical results were presented in this paper.

The morphology of the prepared films was characterized by scanning electron microscopy (SEM) using Quanta 3D FEG, (LEO Electron Microscopy Ltd., Cambridge, England, UK) for the surface of the films before and after modification.

### 2.6. The Release of Rutin from Biopolymeric Film

The release of rutin from the biopolymeric films was studied in a phosphate buffer at pH 5.5, similar to the pH of the human skin. The films were cut into 4 cm^2^ (length × width = 2 cm × 2 cm) pieces, analytically weighted, and then immersed in a 50 mL of phosphate buffer. The temperature during the release process was kept at 35.5 ± 1 °C. After a selected time of release (1; 3; 5; 10; 15; 30; 45; 60; 90, and 120 min), 1.5 mL of the solution was taken for analysis. 

**Figure 2:**
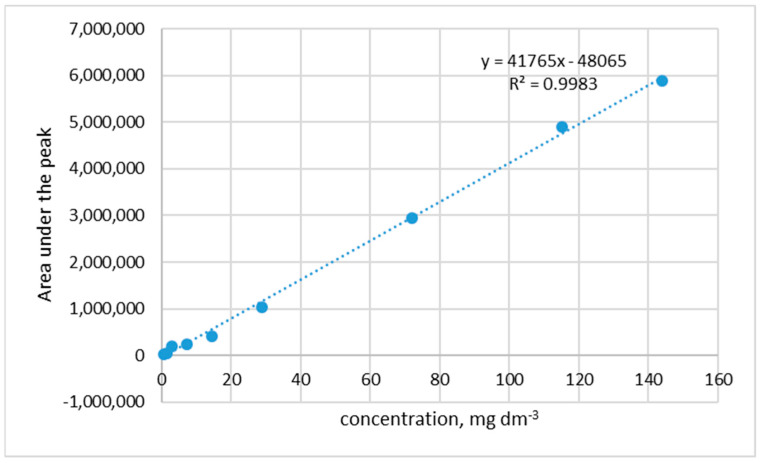
The calibration curve for determination of rutin.

The concentrations of the rutin in 1.5 mL of buffer were measured using the HPLC method with column 250/4.6 Nucleoshel RP 18.5 μm. Before the release measurements, the calibration curve for determining rutin concentration was drawn (Figure 2).

### 2.7. Measurements of Mechanical Properties 

In order to investigate the mechanical properties of CS-GLY-RUT-HA biopolymer films, elasticity analysis was performed using Zwick Roell (Ulm, Germany) under a static load of 10 kg and a crosshead speed of 50 mm/min at room temperature (25 °C). The test was conducted following the ASTM standard method D882 [47]. The elastic modulus was determined as a function of displacement by force applied.

### 2.8. Measurements of Skin Properties 

The measures of skin moisturization and elasticity of skin were determined three times T1 (0 min), T2 (15 min), and T3 (2 h) using the Aramo TS testing machine. Aramo TS is an integrated skin-diagnosis system to analyze various skin parameters. Three women aged between 51 and 59 years were tested.

In the informed consent, all the necessary information about the possible risks, benefits, rights, and obligations related to participation in the study was clearly explained, including the option not to participate. The academic object of the research was reported, maintaining the privacy of the people and the confidentiality of the information collected. Subsequently, a consent document was delivered for the subjects to read and sign. During the testing of formulation, no other cosmetics were used in the places dedicated to the measurements. 

## 3. Results

### 3.1. Structural Studies—Attenuated Total Reflectance Fourier-Transform Infrared Spectroscopy (FTIR-ATR)

**Figure 3:**
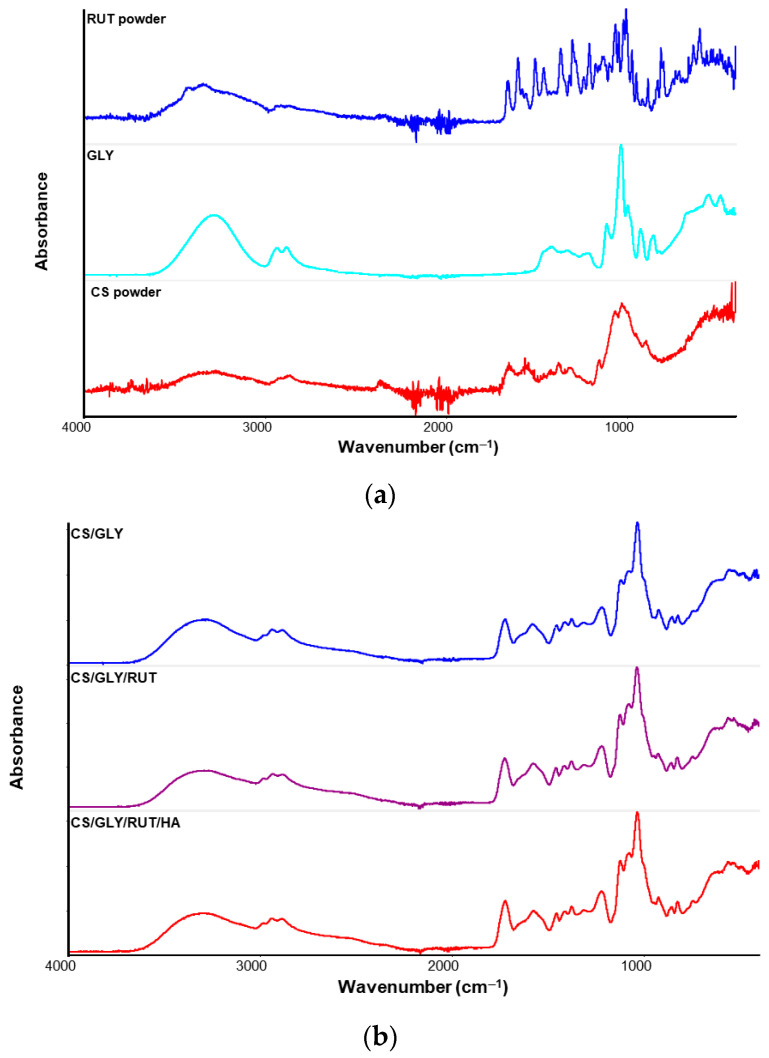
FTIR spectra of pure components (**a**) and prepared films (**b**).

To evaluate the molecular structure of chitosan and chitosan/Rut films, the FTIR-ATR analysis was performed. Additionally, FTIR analysis allows us to observe the presence of functional groups, which can be used to identify compounds and possible interactions between material components. The spectra of pure components and the films obtained have been shown in Figure 3. It can be observed that the position of amide II and amide III does not depend on the addition of rutin to chitosan. However, the amide A and amide B band positions are shifted. It suggests that they participate in the formation of hydrogen bonds between chitosan and rutin. It should be emphasized that the chitosan-based film also contained glycerin and hyaluronic acid. Both of these ingredients were used for obtaining elastic films, flexible enough for cosmetic application, and also for moisturizing properties of the skin after topical applications. Based on our previous research, we added a small amount of hyaluronic acid (HA) to obtain films with good performance and physicochemical properties [47].

### 3.2. Thermal Stability

**Figure 4:**
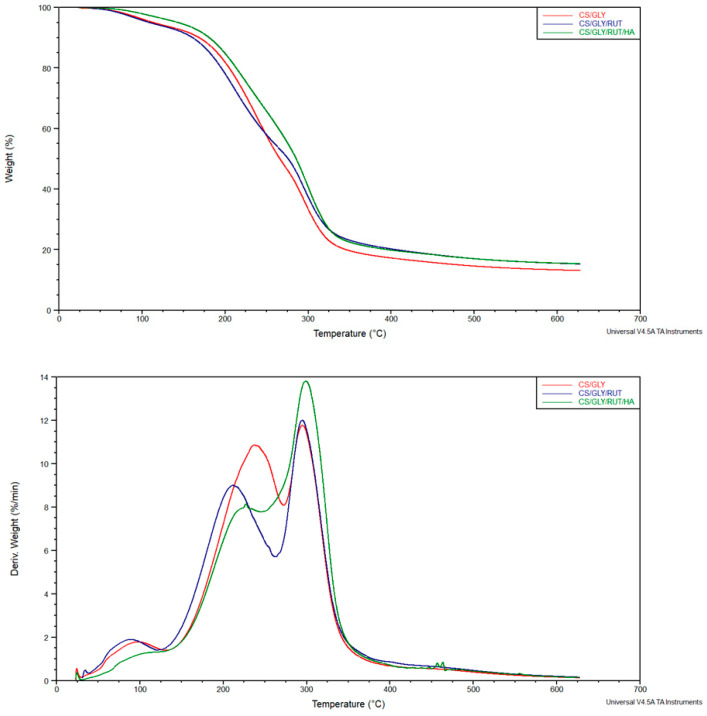
TGA curves of prepared films.

The thermal properties of materials should be considered to know about the possibility of thermal treatment, sterilization, and modification. TGA and DTG curves of chitosan films containing rutin have been shown in Figure 4.

Three regions in DTG curves could be distinguished for CS-GLY, CS-GLY-RUT, and CS-GLY-RUT-HA films. The first one may be correlated with the elimination of water molecules present in the films. The area of a peak in the region responsible for water elimination was much smaller for CS-GLY-RUT-HA films than for CS-GLY and CS-GLY-RUT. Moreover, the process of water elimination started at higher temperatures for CS-GLY-RUT-HA films. The second peak may be correlated with the degradation of materials. It can be seen that the CS-GLY material is more thermally stable than CS-GLY-RUT and CS-GLY-RUT-HA films. The third peak may be correlated with the pyrolysis of the material. The T_max_ (stage 3) for all the materials was comparable, but the peak for CS-GLY-RUT-HA films was the highest. It may suggest that for CS-GLY-RUT-HA films, the water molecules are more strongly bonded with the polymeric film.

### 3.3. Morphology

**Figure 5:**
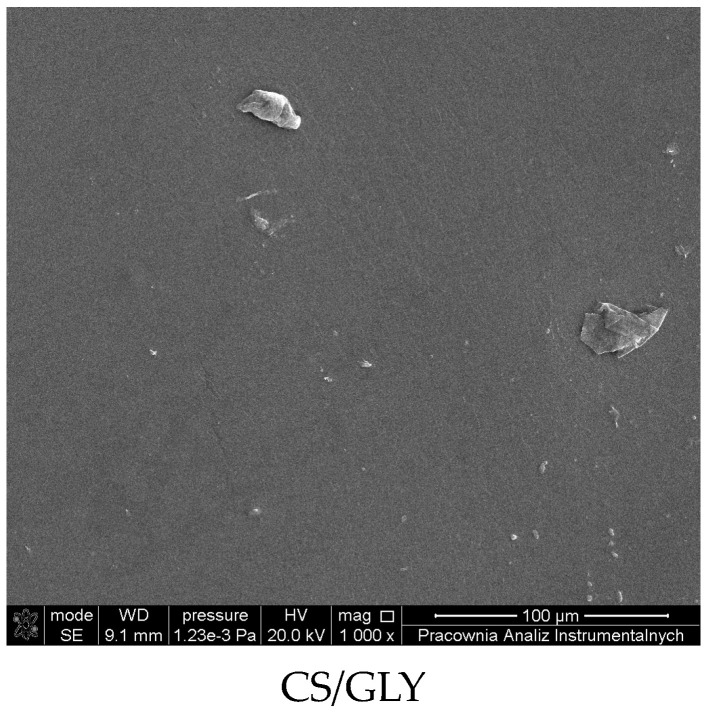

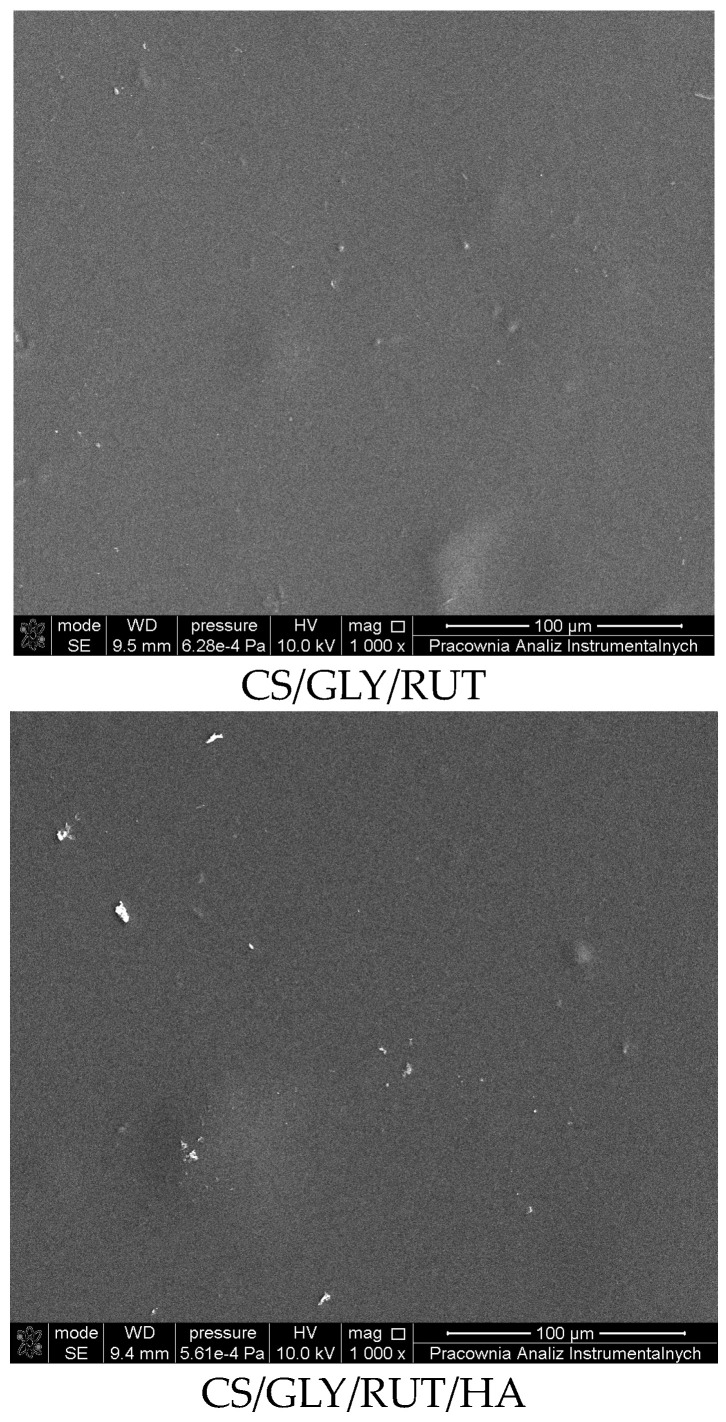
SEM images of CS films.

**Figure 6:**
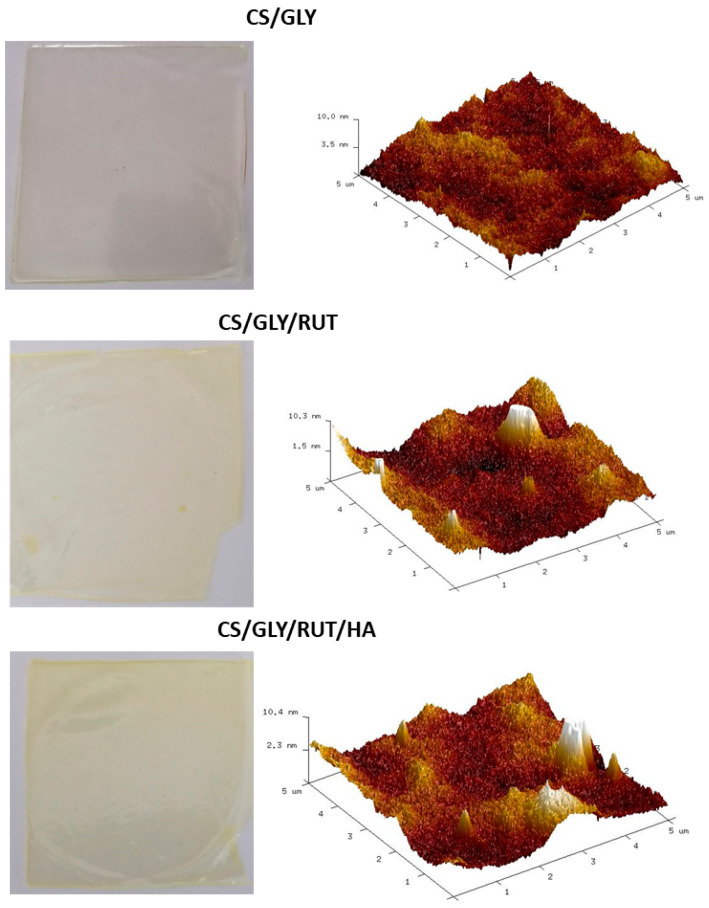
Physical appearance and AFM topography of modified CS films.

The morphology of biopolymer films was investigated by SEM and AFM microscopy. SEM images of the film are shown in Figure 5. AFM imaging has been used to assess the surface properties of the films. The physical appearance of the films and AFM topography of the studied films are shown in Figure 6.

As seen from SEM images, the chitosan films containing glycerin have a flat surface. Glycerin has been added for better film performance and as a moisturizing compound. Moreover, glycerin can enhance the penetration of active substances into the skin. 

Comparing the roughness parameters values for CS-GLY, CS-GLY-RUT, and CS-GLY-RUT-HA films, we can see that the highest roughness was observed for CS-GLY-RUT films. The surface of the CS-GLY-RUT films was characterized by higher parameters of R_q_ (mean square deviation of surface roughness). The roughness of the surface increases after the addition of rutin to chitosan (Table 2).

### 3.4. The Release of Rutin from Biopolymeric Film

**Figure 7:**
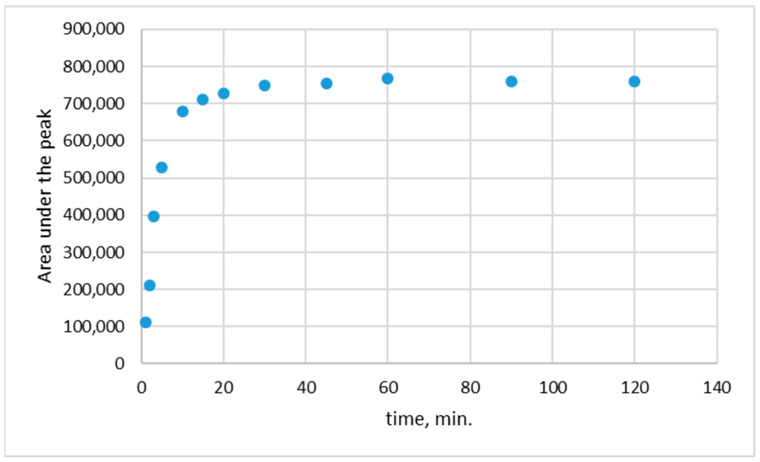
The release of rutin from CS/GLY/RUT film.

**Figure 8:**
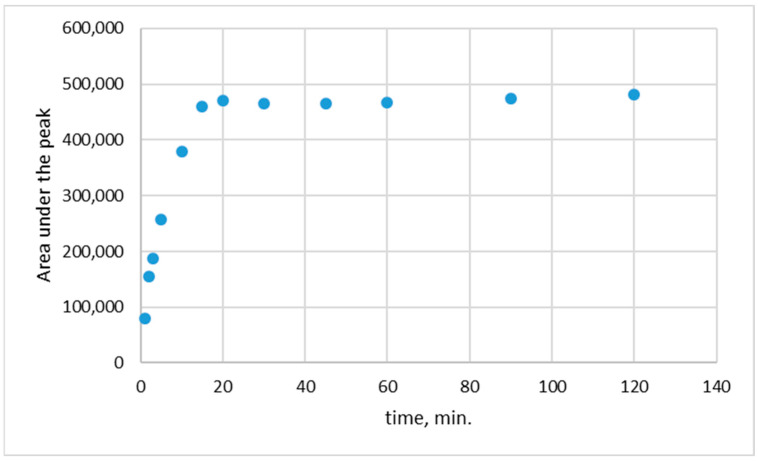
The release of rutin from CS/GLY/RUT/HA film.

The profile of the release of rutin from the biopolymeric film is shown in Figure 7 and Figure 8. In Figure 7, the release of rutin from CS/GLY/RUT films is shown, whereas, in Figure 8, the release from CS/GLY/RUT/HA is presented. 

To explore the in vitro passive release of rutin from the biopolymeric films, the release procedure was conducted in phosphate buffer at pH 5.5. Given that the release behavior of an active substance in a matrix-type drug delivery system is associated with the substance–matrix interaction and the diffusion of the active substance within the matrix material [48,49], our rutin release condition included different types of biopolymeric matrices (different compositions of films).

As can be seen in Figure 7 and Figure 8, the maximum release was found after 20 min at pH 5.5. The average percentage of release from CS/GLY/RUT film was 55.32 ± 1.98%, whereas, from CS/GLY/RUT/HA film, it was 8.33 ± 0.66%. During the release study of rutin from chitosan-based films, it was found that the maximum release was observed after 20 min, and probably this release can also be connected with film solubility in aqueous media. Although chitosan itself is not soluble in water, it is worth emphasizing that in this research, chitosan is present in the form of chitosan lactate, so the release of rutin can also be connected with film solubility in aqueous media. In the presence of HA, the maximum rutin release is smaller. We can conclude that adding HA to formulation leads to stronger bonding of rutin in the chitosan-based films. In hyaluronic acid, there are three most commonly used sites of covalent modification of HA, namely, the carboxylic groups, hydroxyl groups, and –NHCOCH_3_ groups. Moreover, carboxylic groups and hydroxyl groups can form hydrogen bonds. Probably less rutin is released from the chitosan films containing HA because the rutin is additionally bonded by HA.

### 3.5. Mechanical Properties

**Figure 9:**
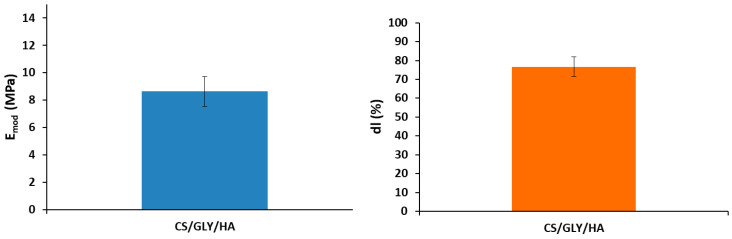
Mechanical properties of chitosan-based films.

For the application of chitosan-based films as a beauty mask, the flexibility and mechanical properties of the film are crucial. In Figure 9, mechanical parameters such as elongation at break and Young’s modulus have been shown. 

As can be seen from Figure 9, elongation at break is above 70%. It shows that the presented biopolymer film based on chitosan with the addition of hyaluronic acid and glycerin is elastic enough to be applied as a cosmetic beauty mask. Yung modulus is also sufficient for cosmetic applications. The flexibility of biopolymer films is crucial in cosmetic applications as consumers expect good quality film on the skin and hair surface. 

### 3.6. Skin Properties

As the aim of this research was the preparation of chitosan film containing rutin for potential cosmetic and biomedical applications, we investigated the properties of the skin after the topical application of chitosan-based films. Two parameters have been compared, skin moisturization and skin elasticity. Three volunteers (females aged 51–59) were chosen for this research. The measurements of elasticity were taken three times: prior to application (T1), 15 min (T2), and 2 h after the application (T3). The results are shown in Table 3. 

The measurement of skin hydration levels is important in terms of different applications and also in terms of future modification of formulations. Most commercial devices for measuring skin hydration are constructed based on electrical-based measurement techniques, particularly the capacitance method. The ARAMO TS 2 device also has a probe that works based on electrical-based measurement techniques, particularly the capacitance method. Measurements of elasticity were obtained using a fixed elasticity probe in the ARAMO TS device. 

## 4. Discussion

Common cosmetics contain moderate amounts of rutin. However, in medical applications, rutin is used due to its potential to reduce the fragility of capillaries. It has been demonstrated that rutin is capable of inhibiting human platelet aggregation stimulated by collagen, decreasing capillary fragility, prolonging activated partial thromboplastin time as well as exerting anti-thrombotic effects. The biological effects of rutin on skin aging have been summarized by Seong Jin Choi et al. [42]. They showed that rutin improved skin dermal density, reduced fine wrinkles, and enhanced elasticity. It has been suggested that rutin may be used as a major ingredient in anti-aging cosmetics in order to improve skin elasticity and reduce wrinkles. In our study, we proposed chitosan films containing glycerin and HA as a new vehicle for rutin. Several polymers have been used so far as vehicles for active substances. However, there are some potential problems that may arise when using polymeric vehicles for controlled delivery systems. These potential problems include cytotoxicity and lack of biocompatibility. In our research, we used biocompatible chitosan and HA as film-forming polymers. These biopolymers do not generate hazardous byproducts upon degradation. So, in our study, we prepared biopolymeric films using biocompatible biopolymers, which are biodegradable, and we incorporated rutin in the films. The very important question to consider is about interactions between HA and chitosan in the solution and later on in the film, which is formed after solvent evaporation. This aspect has been studied by us previously [47]. For a mixture of chitosan with hyaluronic in a solution with different compositions, the miscibility was determined by the viscometric method, and it was found that the blends were partially miscible [50,51]. Both hyaluronic acid and chitosan are polyelectrolytes, in which the properties are strongly associated with the electrostatic interactions that determine the shape of the macromolecule. The macromolecules can be stretched due to the electrostatic repulsive forces between the charges on the functional groups. The carboxylic groups of week HA are mainly in their non-ionized form, whereas the amino groups of chitosan are in ionized form in acidic pH. Thus, the blend of chitosan and hyaluronic acid mainly predominates interactions by hydrogen bonds and repulsive forces. We confirmed that the interactions are mainly due to hydrogen bonds using IR spectroscopy [51,52]. IR spectroscopy is usually done for polymer films, which in the blend can have modified surface properties. Moreover, we found that chitosan films are more polar when hyaluronic acid is added. In this study, we used only a small amount of HA, so it is believed that we have a miscible polymer blend, which can be used for the incorporation of active substances, such as rutin. Rutin is a water-soluble substance, so it can be easily incorporated into cosmetic formulas. However, the release of rutin was more efficient from the chitosan films, which did not contain HA, than from the blend films. Probably the presence of HA leads to interaction between HA and rutin. This may cause less rutin to be released from the matrice. It is also possible that rutin is localized mainly on the surface of the film. If rutin is localized on the surface, it is good from a cosmetic point of view because more of this active substance can be released after application on the skin, and the fragility of capillaries can be reduced even more. 

In our study, the elasticity increased only slightly after the topical application of chitosan modified by glycerol. When we added rutin to chitosan and HA, the elasticity was not improved; it remained at the same level. However, after topical application of the films, especially after a short time after film removal, we observed an increase in skin moisturization. The beauty mask proposed by us was designed for application for no longer than 30 min, and during that time, chitosan cannot degrade. However, after usage, when the mask is wasted, the chitosan mask is biodegradable. Moreover, in our research, we used lactic acid (LA) to dissolve chitosan. LA is a safe ingredient in cosmetic formulations. It is widely used for pH modification in cosmetic formulas. Chitosan itself is not soluble in water, so acidic media were needed to obtain a soluble form of chitosan and form a good quality beauty mask. Chitosan lactate can be soluble in water, so the release of rutin in our research can also be connected with chitosan film disintegration and dissolution. 

It is worth emphasizing that chitosan has been loaded with other active substances, for example, honey [53], curcumin [54,55], plant essential oil [56], and many other compounds [8,57]. Chitosan-based membranes and nanoparticles can be used in drug delivery, per-oral delivery, pulmonary drug delivery, nasal drug delivery, mucosal drug delivery, gene delivery, buccal drug delivery, vaccine delivery, vaginal drug delivery, and cancer therapy [58].

## 5. Conclusions

Chitosan–rutin films and chitosan–HA–rutin films have been successfully obtained and can be proposed as beauty masks. The addition of rutin to chitosan did not have an influence on the chitosan structure but changed the thermal stability. Chitosan films containing glycerol were more thermally stable than chitosan films containing glycerol, hyaluronic acid, and rutin. The roughness of the films was bigger for the films containing rutin.

The maximum rutin release was found after 20 min at pH 5.5. The average percentage of release was bigger for chitosan film containing rutin than for chitosan/hyaluronic acid film containing rutin. Skin elasticity was not changed after topical application of the films, whereas skin moisturization was a little improved after 15 min. After the topical application of the proposed chitosan–rutin mask, the appearance of the skin was improved. However, the research has been done only on the group of three females, so further studies should be proposed. Nevertheless, the chitosan-based film showed the potential for delivery of rutin and can be considered a vehicle for this active substance. The addition of hyaluronic acid somewhat decreased the release of rutin, but both films have the potential for cosmetic applications.

## Figures and Tables

**Table 1:** Ingredients of the chitosan-based beauty mask.

Ingredients	Amount (%) CS/GLY/RUT	Amount (%) CS/GLY/RUT/HA
GLY	1.08	1.23
CTS	1.97	1.93
HA	0	0.02
LA	2.67	2.67
RUT	0.02	0.02
aqua	to 100	to 100

**Table 2:** The roughness parameter (R_q_) and thickness (Th) of chitosan films with different compositions.

Sample	R_q_ (nm)	Th (mm)
CS/GLY	1.07 ± 0.05	0.066 ± 0.004
CS/GLY/RUT	2.51 ± 0.17	0.073 ± 0.006
CS/GLY/RUT/HA	1.87 ± 0.27	0.087 ± 0.005

**Table 3:** Skin properties: moisturization and elasticity measured by Aramo TS2.

Sample	Moisturization			Elasticity		
	T1	T2	T3	T1	T2	T3
CS/GLY	38	42	39	63	66	66
CS/GLY/RUT	38	42	40	61	61	57
CS/GLY/RUT/HA	37	42	40	60	59	57

## Data Availability

All data have been shown in the paper.
